# Proteogenomic Evaluation of Cerebrospinal Fluid in Alzheimer's Disease

**DOI:** 10.1002/alz.086066

**Published:** 2025-01-09

**Authors:** Raquel Puerta, Pablo García‐González, Itziar de Rojas, Clàudia Olivé, Fernando García‐Gutiérrez, Oscar Sotolongo‐Grau, Bart Smets, Asif Emon, Yun Ju Sung, Ruth Frikke‐Schmidt, Natalie L Marchant, Jean‐Charles Lambert, Sergi Valero, Marta Marquié, Alfredo Ramirez, Carlos Cruchaga, Victoria Fernández, Amanda Cano, Mercè Boada, Alfredo Cabrera Socorro, Agustin Ruiz

**Affiliations:** ^1^ Ace Alzheimer Center Barcelona – International University of Catalunya (UIC), Barcelona Spain; ^2^ CIBERNED, Network Center for Biomedical Research in Neurodegenerative Diseases, National Institute of Health Carlos III, Madrid Spain; ^3^ Ace Alzheimer Center, Barcelona Spain; ^4^ Janssen Research & Development, A Division of Janssen Pharmaceutica, Neuroscience Therapeutic Area, Beerse Belgium; ^5^ NeuroGenomics and Informatics Center, Washington University School of Medicine, St. Louis, MO USA; ^6^ Hope Center for Neurological Disorders, St. Louis, MO USA; ^7^ Department of Clinical Biochemistry, Copenhagen University Hospital ‐ Rigshospitalet, Copenhagen Denmark; ^8^ Division of Psychiatry, University College London, London UK; ^9^ Univ. Lille, Inserm, CHU Lille, Institut Pasteur de Lille, U1167‐RID‐AGE Facteurs de risque et déterminants moléculaires des maladies liées au vieillissement, F‐59000, Lille France; ^10^ Inserm U1167 ‐ Université de Lille – CHU de Lille – Institut Pasteur de Lille – LabEx DISTALZ, Lille France; ^11^ Division of Neurogenetics and Molecular Psychiatry, Department of Psychiatry and Psychotherapy, Faculty of Medicine and University Hospital Cologne, University of Cologne, Cologne Germany; ^12^ Cluster of Excellence Cellular Stress Responses in Aging‐Associated Diseases (CECAD), University of Cologne, 50931, Cologne Germany; ^13^ Department of Psychiatry & Glenn Biggs Institute for Alzheimer’s and Neurodegenerative Diseases, San Antonio, TX USA; ^14^ Deutsches Zentrum für Neurodegenerative Erkrankungen e. V. (DZNE) Bonn, Bonn Germany; ^15^ NeuroGenomics & Informatics Center, Washington University School of Medicine, St. Louis, MO USA; ^16^ Hope Center for Neurological Disorders, Washington University School of Medicine, St. Louis, MO USA

## Abstract

**Background:**

Alzheimer's disease (AD) is a complex disorder with a strong genetic component, yet many genetic risk factors remain unknown. Integrating genome‐wide association studies (GWAS) and high‐throughput proteomic platforms is a useful strategy to evaluate protein quantitative trait loci (pQTLs) and to detect candidate genes and pathways involved in AD. Due to the novelty of these techniques, the identification of reliable protein measures through a comprehensive quality control is mandatory.

**Method:**

We researched the cerebrospinal fluid (CSF) proteome using the SOMAscan 7k (7289 proteins), and the Olink Explore (2943 proteins) platform in the Ace Alzheimer Center Barcelona CSF Cohort. To assess reproducibility and reliability of proteomic measures, we analysed 1) intra‐platform correlation of two independent SOMAscan assays using two aliquots of the same sample, and 2) inter‐platform correlation between SOMAscan and Olink techniques. We also explored associations of highly reliable proteins with the AD Polygenic risk score (PRS) and the conversion to AD dementia. Additionally, we have performed an extensive pQTLs mapping of the SOMAscan platform and analysed pQTLs of significant proteins linked to disease progression.

**Result:**

Our results revealed 2469 proteins with high intra‐platform correlation (rho≥0.5), selected as candidates for further analysis. Moreover, over 600 proteins demonstrated strong inter‐platform correlation, reinforcing their robustness. Extending our analysis to the entire CSF cohort (n=1322), we identified 85 proteins (Padj.<0.05) associated with the AD‐PRS. Additionally, 93 significant proteins were linked with conversion to AD dementia. Strikingly, these conversion‐associated proteins exhibited 89 genome‐wide significant pQTLs in the genome, with more than 47% of the genetic signals localized within the APOE‐rs429358 locus.

**Conclusion:**

Our findings emphasize the importance of reliability assessment of highly multiplexed proteomic panels. Using robust measures, we identified significant proteins related to AD genetic risk and disease progression. These insights deepen the AD pathogenesis understanding, expand future research and therapeutic interventions.

